# Antibacterial and Antifungal Tannic Acid Coating on Plasma-Activated Titanium Alloy Surface

**DOI:** 10.3390/ijms26157051

**Published:** 2025-07-22

**Authors:** Mariusz Winiecki, Magdalena Stepczyńska, Maciej Walczak, Ewelina Soszczyńska, Magdalena Twarużek, Dorota Bociaga, Marek Trzcinski, Marta Michalska-Sionkowska, Krzysztof Moraczewski

**Affiliations:** 1 Department of Constructional Materials and Biomaterials, Faculty of Materials Engineering, Kazimierz Wielki University, 30 Chodkiewicza Street, 85-064 Bydgoszcz, Poland; 2 Department of Polymer Materials Engineering, Faculty of Materials Engineering, Kazimierz Wielki University, 30 Chodkiewicza Street, 85-064 Bydgoszcz, Poland; kmm@ukw.edu.pl; 3 Department of Environmental Microbiology and Biotechnology, Faculty of Biological and Veterinary Sciences, Nicolaus Copernicus University, 1 Lwowska Street, 87-100 Torun, Poland; maciej.walczak@umk.pl (M.W.); mms@umk.pl (M.M.-S.); 4 Department of Physiology and Toxicology, Faculty of Biological Sciences, Kazimierz Wielki University, 30 Chodkiewicza Street, 85-064 Bydgoszcz, Poland; eweso@ukw.edu.pl (E.S.); twarmag@ukw.edu.pl (M.T.); 5 Institute of Materials Science and Engineering, Faculty of Mechanical Engineering, Lodz University of Technology, 1/15 Stefanowskiego Street, 90-537 Lodz, Poland; dorota.bociaga@p.lodz.pl; 6 Division of Surface Science, Faculty of Chemical Technology and Engineering, Bydgoszcz University of Science and Technology, 7 Prof. S. Kaliskiego Avenue, 85-796 Bydgoszcz, Poland; marek.trzcinski@pbs.edu.pl

**Keywords:** antibacterial, antifungal, tannic acid, coatings, titanium alloy, surface properties, surface modification

## Abstract

Titanium (Ti) alloys, renowned for their exceptional physicochemical properties and high biocompatibility, are widely utilized in orthopedic and dental implants; however, their lack of intrinsic antimicrobial activity significantly increases the risk of implant-associated infections, often leading to severe complications and implant failure. Developing antimicrobial coatings on Ti implants is therefore a promising strategy. In this study, tannic acid (TA) coatings were deposited by immersing Ti alloy surfaces—beforehand activated by low-temperature oxygen plasma—in TA solutions at 2, 5, and 8 wt%. Coatings were characterized by scanning electron microscopy (SEM), X-ray photoelectron spectroscopy (XPS), water contact angle (WCA) measurements, and Folin–Ciocalteu release assays, and their cytocompatibility and antimicrobial performance were assessed in vitro. Surface characterization confirmed the formation of uniform TA layers, and WCA measurements indicated enhanced hydrophilicity relative to unmodified Ti (82.0° ± 3.6°), with values decreasing as TA concentration increased (from 35.2° ± 3.2° for 2% TA to 26.6° ± 2.8° for 8% TA). TA release profiles exhibited an initial burst followed by sustained diffusion, with 5% and 8% coatings releasing significantly more TA than 2% coatings. Coatings containing ≥ 5% TA demonstrated bactericidal activity—achieving > 2-log_10_ reductions—against *Escherichia coli*, *Staphylococcus aureus*, and *Pseudomonas aeruginosa*, and also showed inhibitory effects against *Candida albicans*. Importantly, all coatings remained cytocompatible with NIH/3T3 fibroblasts, and the released tannic acid hydrolysis products (particularly gallic acid) enhanced their proliferation. These findings indicate that plasma-activated titanium surfaces coated with ≥5 wt% tannic acid impart broad-spectrum antimicrobial efficacy and hold potential to reduce implant-associated infections and improve long-term outcomes in orthopedic and dental applications.

## 1. Introduction

Titanium and its alloys are widely used in orthopedic and dental implants due to their excellent physicochemical properties and high biocompatibility with host tissues, which promote predictable long-term treatment outcomes [1]. However, titanium-alloy implants do not possess inherent antimicrobial activity. Once exposed to the external environment, their surfaces become susceptible to microbial adhesion and biofilm formation, triggering local inflammation in surrounding tissues. This, in turn, impairs early osseointegration and often leads to implant failure [2,3].

The most frequent pathogens responsible for implant-related infections are Gram-negative *Escherichia coli* and Gram-positive *Staphylococcus aureus* [4]. Of particular concern are bacteria from the so-called ESKAPE panel (*Enterococcus faecium*, *Staphylococcus aureus*, *Klebsiella pneumoniae*, *Acinetobacter baumannii*, *Pseudomonas aeruginosa*, and *Enterobacter* species), which readily form biofilms and exhibit resistance to conventional antibiotic therapies [5]. Biofilm formation isolates the inflammatory environment, suppresses the host immune response, and facilitates bacterial colonization at the bone–implant interface and in adjacent tissues [6].

In dental applications, peri-implantitis arises when oral pathogens invade the interface between the gingival soft tissue and the implant [7]. In addition to bacteria, the oral cavity harbors the yeast *Candida albicans* and related species, which play pivotal roles in multispecies biofilms [8,9]. *Candida albicans*, a commensal and opportunistic pathogen, is a frequent cause of fungal infections—especially in immunosuppressed patients—and can act as a bridge for bacterial adhesion [10].

To minimize the risk of implant-associated infections, numerous surface-modification strategies have been explored [11,12,13,14]. A common approach is to coat titanium implants with biodegradable polymers—such as alginate, collagen, cellulose, gelatin, chitosan, hyaluronic acid, or polydopamine—or with polyphenols (e.g., tannic acid, gallic acid, pyrogallol) [15]. Such coatings can also serve as carriers for antibiotics, metal-oxide nanoparticles, or antimicrobial peptides [12].

Tannic acid, a natural polyphenol, has garnered attention in biomedical engineering due to its broad spectrum of bioactivities [16], including antibacterial, antioxidant, antiviral, antifungal, and antimutagenic effects [17,18]. It exhibits excellent biocompatibility and readily forms stable coatings on diverse substrates [19,20,21,22,23,24,25,26,27]. Beyond its intrinsic activity, tannic acid can act as a versatile carrier for drugs and nanoparticles, enabling multifunctional implant surfaces. For instance, tannic acid coatings have been used to deliver gentamicin for infection prophylaxis [28], magnesium or strontium ions for osteoimmunomodulation [25,29], and hydroxyapatite for bone regeneration [30]. Such multifunctional layers have improved the performance and safety of both orthopedic [31] and dental implants [32].

Tannic acid adsorbs onto hydrophilic surfaces primarily through hydrogen bonding and can be rapidly deposited onto metal substrates [33,34]. Notably, low-temperature oxygen plasma treatment of titanium alloys, as proposed in our previous work, significantly increases the surface density of hydroxyl groups, thereby enhancing the adsorption of tannic acid. This results in coatings with improved uniformity, stronger adhesion to the substrate, and greater thickness compared to those formed on untreated titanium alloy surfaces [35].

The aim of this study is to develop and characterize a tannic-acid coating on low-temperature oxygen-plasma-treated titanium alloy surfaces, and to investigate its antibacterial and antifungal efficacy.

## 2. Results and Discussion

### 2.1. Surface Characterization

**Figure 1:**
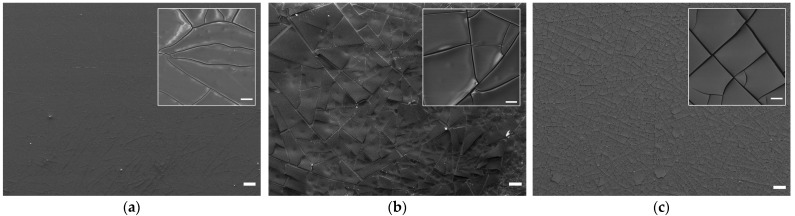
SEM images of coatings on titanium alloy discs: (**a**) Ti-TA2, (**b**) Ti-TA5, and (**c**) Ti-TA8 at 50× magnification; insets: 1000× magnification; bar: 10 μm.

**Figure 2:**
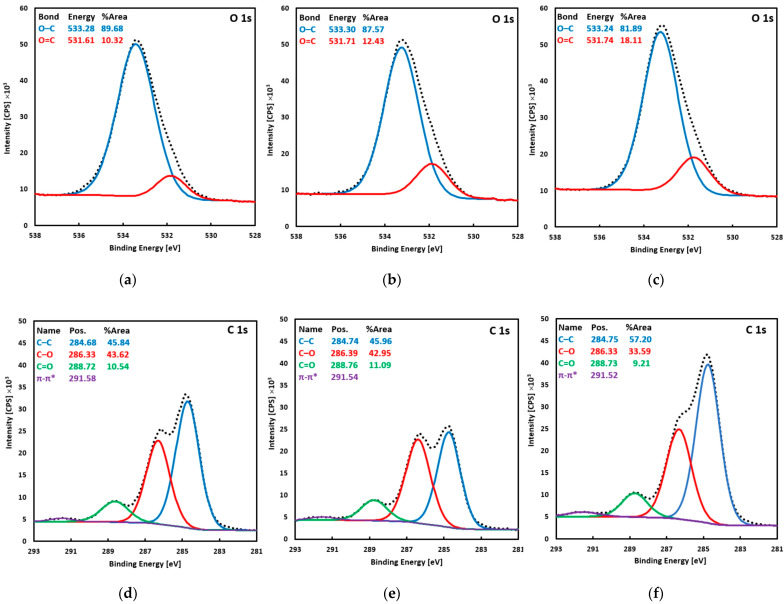
XPS spectra of the O 1s region (**a**–**c**) and the C 1s region (**d**–**f**) recorded for the Ti-TA2 (**a**,**d**), Ti-TA5 (**b**,**d**), and Ti-TA8 discs (**c**,**f**).

The surface morphology of the Ti-TA2, Ti-TA5, and Ti-TA8 discs is shown in the SEM images in Figure 1. The chemical composition of the surface of the Ti-TA2, Ti-TA5, and Ti-TA8 discs, gained from the XPS measurements, is listed in Table 1, while Figure 2 depicts the XPS spectra of the O 1s region (Figure 2a–c) and the C 1s region (Figure 2d–f).

Based on the SEM images, it is evident that the experimental discs are uniformly coated with tannic acid. The coating morphology is heterogeneous, characterized by numerous cracks that define flat-plate–like structures. These plate-like features appear predominantly as elongated, polygonal, or quadrilateral shapes on the Ti-TA2, Ti-TA5, and Ti-TA8 samples, respectively. In the Ti-TA2 and Ti-TA5 specimens, the coatings exhibit occasional protrusions and bulges along the cracks—features absent in the Ti-TA8 sample. SEM observations further suggest that, for all discs, the tannic acid coatings adhere well to the titanium substrate. However, in the Ti-TA5 and Ti-TA8 samples, slight deformations observed at the edges of individual plates may indicate the onset of delamination. The comparatively smoother microstructure observed on the Ti-TA2 surface suggests a thinner coating concerning the other samples. This is likely associated with a lower tannic acid concentration, as supported by the visibility of substrate artifacts beneath the coating. In contrast, the Ti-TA5 and Ti-TA8 samples do not show distinct features that correlate directly with coating thickness.

For all samples, the survey spectra showed only carbon and oxygen; no other elements were detected. At the C 1s level, four characteristic components of tannic acid are observed. The peak at ≈284.7 eV, attributed to C–C and C–H bonds, has the highest intensity (45.8–57.2% of the C 1s area). At ≈286.3 eV, a peak corresponding to C–O bonds (phenolic C–OH and ether linkages) accounts for 22.6–43.6% of the C 1s area. At ≈288.7 eV (≈10%), an O–C=O feature is assigned to carbonyl/carboxyl groups. Finally, at ≈291.5 eV, a weak π–π* shake-up satellite peak of aromatic rings appears; this peak was excluded when determining atomic concentrations. The O 1s envelope was fitted with two components: a dominant peak at ≈533.3 eV (81.9–89.7% of the O 1s area), corresponding to oxygen in C–O–C and C–OH environments (confirming that hydroxyl and ether functionalities predominate, and a smaller peak at ≈531.7 eV, attributed to O=C bonds in carbonyl/carboxyl environments.

**Figure 3:**
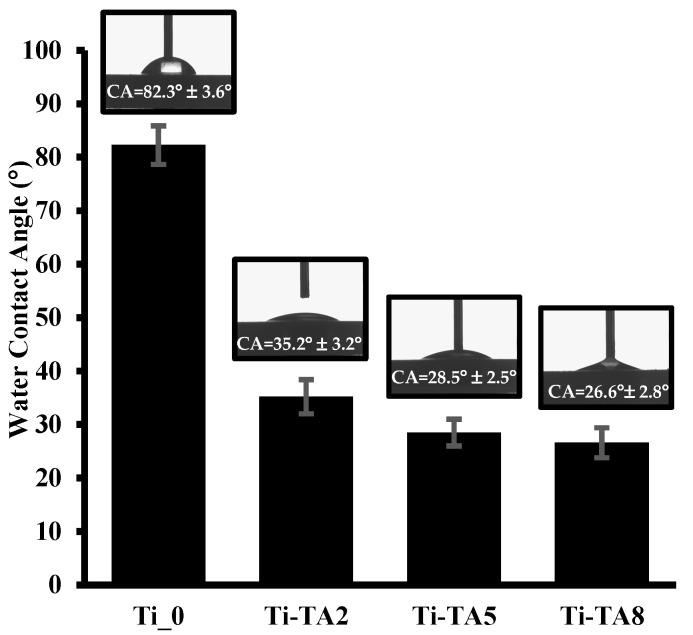
Water contact angles of Ti_0 and tannic acid modified discs (Ti-TA2, Ti-TA5, Ti-TA8).

The contact angle measurements for Ti-TA2, Ti-TA5, and Ti-TA8 versus unmodified Ti_0 discs are shown in Figure 3.

As shown in Figure 3, the unmodified Ti_0 surface exhibits a contact angle of 82.3° ± 3.6°, indicating a weakly hydrophilic character. Upon tannic acid deposition, the contact angle decreases significantly, reflecting enhanced hydrophilicity. Specifically, the Ti-TA2 surface shows a contact angle of 35.2° ± 3.2°, corresponding to moderate hydrophilicity, while higher TA concentrations yield contact angles of 28.5° ± 2.5° (Ti-TA5) and 26.6° ± 2.8° (Ti-TA8), indicative of strongly hydrophilic surfaces. This trend arises because tannic acid’s numerous hydroxyl groups interact readily with water. This trend arises because tannic acid’s numerous hydroxyl groups interact readily with water, and higher TA loadings increase the density of hydrophilic groups, thereby reducing the water contact angle.

### 2.2. Tannic Acid Release

**Figure 4:**
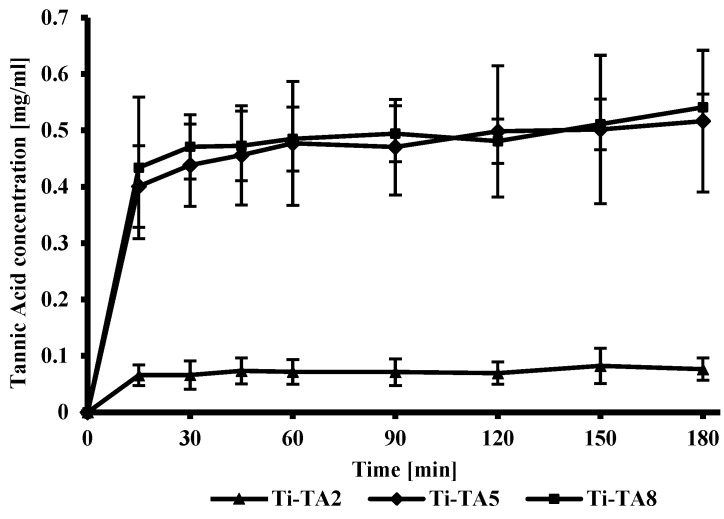
In vitro release profile of tannic acid from the surface of titanium discs.

Figure 4 illustrates the in vitro release profiles of tannic acid from Ti-TA2, Ti-TA5, and Ti-TA8 discs. The release kinetics for Ti-TA5 and Ti-TA8 exhibit similar trends, with both samples exhibiting significantly higher release rates compared to Ti-TA2. This behavior can be attributed to the higher initial loading of tannic acid and the thicker coating layer present in the Ti-TA5 and Ti-TA8 samples. All samples display an initial burst release, a common phenomenon associated with the rapid diffusion of surface-associated compounds. Burst effect for tannic acid coatings was observed by Kaczmarek-Szczepańska et al. [19], and fast initial release of polyphenols from gelatin films was observed by Liu et al. [36]. Other studies have shown that the release profile may be changed by incorporating polyphenols into matrices, and adjusting crosslinker concentration can modulate the release profile [37]. In this study, tannic acid release was governed by polymer solubility and the diffusion into the PBS buffer.

### 2.3. Cytocompatibility Studies

**Figure 5:**
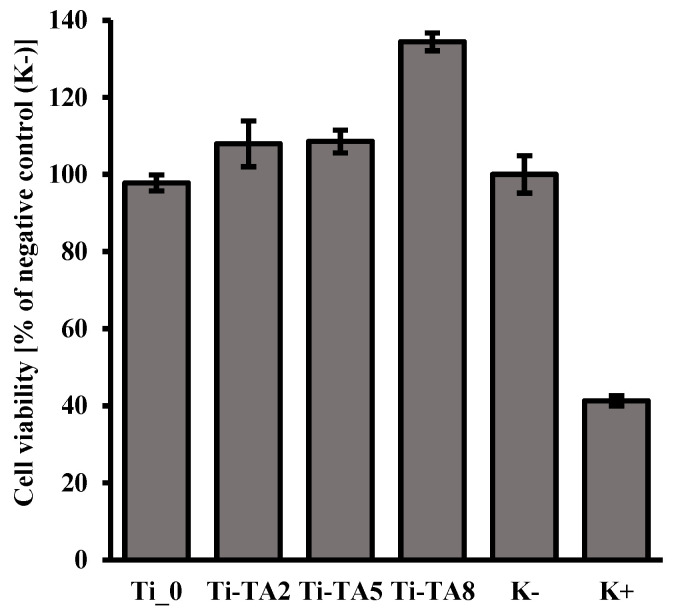
The results of the MTT test for the assessment of cell viability of the NIH/3T3 fibroblast cells after 24 h of incubation with 100% extracts obtained from samples Ti_0 and tannic acid modified (Ti-TA2, Ti-TA5, Ti-TA8). Reference to negative control (K−). K+—positive control.

According to the ISO 10993-5 standard [38], the material is considered non-toxic if cell viability exceeds 70%. In the case of the tested materials, all samples showed this level of viability after 24 h of incubation for 100% of the extracts (Figure 5). Therefore, it can be considered that these materials are safe for in vitro contact. For all tannic acid-coated samples (Ti-TA2, Ti-TA5, Ti-TA8), an effect of increased proliferation level was observed compared to the culture in the medium without any additives (K−). These findings align with literature reports that polyphenols promote fibroblast growth on surfaces [16,39]. According to literature reports, tannic acid itself does not necessarily directly stimulate fibroblast proliferation, but its hydrolysis products (especially gallic acid) can significantly improve the conditions for the growth and multiplication of these cells [40].

Ti-TA8 shows a higher increase in cell proliferation compared to Ti-TA2 and Ti-TA5, which is probably the result of the increased amount of gallic acid in the extract, which was obtained as a result of 24 h contact of the coating with the culture medium. The study of tannic acid release (Figure 4) showed that after 180 min for sample Ti-TA8, the amount of released tannic acid is the highest. After around 24 h, it is released more in comparison to the samples Ti-TA2 and Ti-TA5.

Taking into account the results of the XPS study (Table 1), which showed that for samples Ti-TA2 and Ti-TA5 the level of dominant phenolic C–O and aromatic C–C signals is at a similar level and increases for sample Ti-TA8, it can be concluded that in the extract obtained after 24 h of contact we have an increased content of gallic acid (phenolic compound) which is a product of hydrolysis of tannic acid. As a result, we observe increased proliferation of fibroblast cells for sample Ti-TA8 (Figure 5).

### 2.4. Antibacterial Activity

The results of the number of test microorganisms capable of growth after the time of contact with the tested material are presented in Table 2.

The Ti_0 and Ti-TA2 showed no bactericidal effect. In contrast, Ti-TA5 and Ti-TA8 exhibited a >2-log_10_ reduction in bacterial counts, meeting the ISO 22196 standard [41] criteria for bactericidal activity.

**Figure 6:**
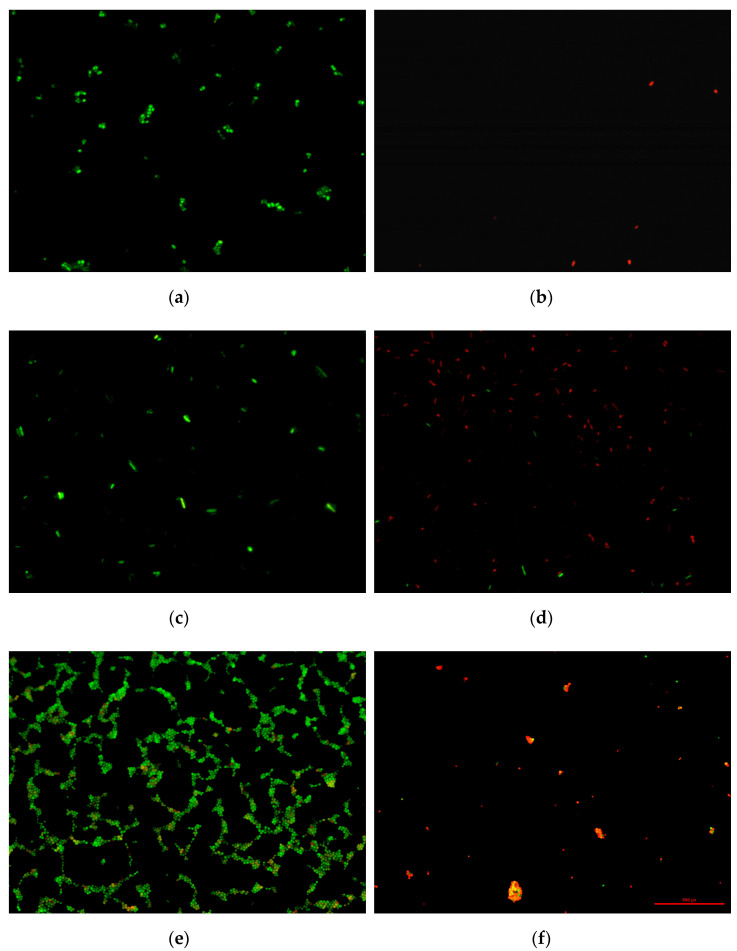
LIVE/DEAD staining of bacteria: *E. coli* from Ti_0 (**a**) and *E. coli* from Ti-TA5 (**b**), *P. aeruginosa* from Ti_0 (**c**) and *P. aeruginosa* from Ti-TA5 (**d**), *S. aureus* from Ti_0 (**e**), and *S. aureus* from Ti-TA5 (**f**).

Figure 6 shows photographs from microscopic analyses of the effect of the tested materials on cell survival. For all tested bacterial strains on titanium plates not covered with tannic acid (Ti_0), no bactericidal effect was noted. There is confirmation of the results obtained in the culture test in accordance with the ISO standard. On the other hand, at the 5% concentration of tannic acid used on Ti-TA5 discs, a lethal effect was obtained in relation to the tested bacteria. This result confirms the culture and normative studies.

The cells in the photographs are red, which indicates damage to their cell membranes and leakage of cell content. Moreover, in the case of *E. coli*, not only was a change in the color of the cells observed, but also a radical reduction in their number in the field of view. This proves that under the influence of tannic acid, first the cell covers were damaged, and then the cells were completely lysed (dissolved). This effect was not observed for *S. aureus* and *P. aeruginosa*. However, this does not change the fact that the Ti-TA5 material showed bactericidal activity against all tested bacterial strains.

### 2.5. Antifungal Activity

The results of the number of *Candida albicans* capable of growth after the time of contact with the tested material are presented in Table 3.

Tannic acid coatings on Ti-TA5 and Ti-TA8 discs were found to have an inhibitory effect on the growth of *Candida albicans*. The inhibitory effect was not found for the coating on Ti-TA2. After 24 h of exposure, TA induced a reduction in cell viability. There were significant effects in relation to the control. After six hours of incubation, no such effect was detected. In the case of concentrations Ti-TA5 and Ti-TA8, a decrease of two orders of magnitude in the number of yeast cells was noted.

Tannic acid and tannins have been found to be toxic to fungi, bacteria, and viruses, and to inhibit their growth. This is consistent with the results of the present study, which showed that tannic acid inhibits the growth of *C. albicans*. The inhibitory activity of tannic acid on the growth and acid production of *C. albicans* suggests that tannic acid could play an important role in the prevention and treatment of oral candidiasis [9,42,43]. Tannic acid can also potentiate the antifungal effects of azole antifungals against some Candida species, suggesting a synergistic effect [43].

The antimicrobial activity of tannic acid against fungi, bacteria, and viruses results from its multifaceted effects on microbial structures and cellular processes [17,44,45]. One of the primary mechanisms involves its ability to denature proteins through the formation of complexes with functional groups, leading to the loss of enzymatic and structural activity. Tannic acid can also disrupt the integrity of cell membranes by interacting with lipid components, leading to increased permeability, destabilization, and ultimately, cell death. Another important aspect is its capacity to chelate metal ions essential for the proper function of many microbial enzymes, thereby inhibiting key metabolic processes [44,45]. Furthermore, tannic acid can directly inhibit the activity of enzymes critical to pathogen survival and replication, such as polymerases, oxidases, and hydrolases. It may also interact with nucleic acids, impairing the replication and transcription of genetic material, which is particularly significant in the case of viruses [17,44]. Under certain conditions, tannic acid may exert pro-oxidant effects, generating reactive oxygen species that damage cellular structures [37]. The combined action of these mechanisms underlies the broad-spectrum antimicrobial efficacy of tannic acid against various pathogenic organisms [17,44,45,46].

## 3. Materials and Methods

### 3.1. Sample Modification

Samples in the shape of discs 8 mm in diameter and 1.5 mm in thickness were machined from a Ti-6Al-4V (Grade 5) rod (Bibus Metals, Dąbrowa, Poland). The discs were polished with abrasive SiC paper (600, 1200, and 2000 grids), then underwent ultrasonic cleaning in acetone for 15 min and dried at room temperature. Subsequently, discs were subjected to low-temperature plasma (LTP) treatment according to the procedure developed in previous work [35]. LTP treatment was performed using the Femto Plasma Generator (Diener electronic GmbH & Co. KG, Ebhausen, Germany) with a nominal power of 100 W. The discs were placed in a plasma generator chamber on a metal slab and exposed to plasma discharge. The samples were treated at room temperature with an O_2_ plasma (plasma power of 80 W) for 10 min with a gas flow rate of 15 mL/min and a chamber pressure of 0.1–1 mbar.

Subsequently, surface modification of the samples specified above with tannic acid coatings was performed. In the modification process, tannic acid C_76_H_52_O_46_ with a molecular weight of 1701.20 g/mol (Merck KGaA, Darmstadt, Germany) was used. Tannic acid solutions were prepared in 10 mM TRIS-HCl buffer (Chempur, Piekary Śląskie, Poland) with a pH of 8.5 of three different concentrations (2%, 5% and 8%). The Ti discs were immersed in the prepared solutions and left for 24 h at room temperature to deposit the tannic acid coating on the surface of the titanium discs. After 24 h, the discs were removed from the acid solutions and dried for 24 h at 40 °C. Depending on the concentration of the tannic acid solutions used, the samples are referred to as Ti-TA2, Ti-TA5, and Ti-TA8. Where applicable, polished Ti-alloy samples were used as the control and are referred to as Ti_0.

### 3.2. Sample Characterization

A scanning electron microscope (SEM) (Hitachi SU8010, Hitachi High-Technologies Co., Tokyo, Japan) was applied to examine the surface morphology of the samples. Observations were performed with the secondary electrons (SEs) mode at an accelerating voltage of 5 kV to capture the surface images with a magnification of 50× and 1000×.

X-ray photoelectron spectroscopy (XPS) analysis was performed using a VG-Scienta R3000 analyzer (Scienta Omicron, Uppsala, Sweden). The excitation source was the RS 40B1 lamp (Prevac Sp. z o.o., Rogów, Poland) emitting AlKα (1486.6 eV) line. The photoelectron take-off angle was 90°. High-resolution spectra were recorded with an energy step of ΔE = 100 meV. The operating pressure in the analysis chamber was maintained below 5 × 10^−10^ mbar. Spectral deconvolution was conducted using CasaXPS software (v.2.3.16—Casa Software Ltd., Teignmouth, UK), modeling a Shirley-type background.

Contact angle measurements of the discs’ surfaces were conducted in triplicate, with each measurement repeated three times, using a DSA 100 Goniometer (A. KRÜSS Optronic GmbH, Hamburg, Germany) equipped with an automated liquid drop dosing system. A 4 µL droplet of distilled water was dispensed onto the surface of each tested disc. The contact angle was recorded from both the left and right sides of the droplet. Three measurements were obtained per sample, and the final contact angle value was calculated as the mean of all individual readings.

### 3.3. In Vitro Studies of the Tannic Acid Release

The release profile of tannic acid from titanium discs was evaluated using the Folin–Ciocalteu assay, a widely employed technique for quantifying total phenolic content [47,48]. Samples were individually placed into wells of a 12-well plate, each containing 2 mL of phosphate-buffered saline (PBS, pH 7.4), and incubated at 37 °C. Sampling was carried out at specified intervals: 15, 30, 45, 60, 90, 120, 150, and 180 min. At each time point, a 20 µL aliquot was taken from the supernatant, diluted with distilled water to a final volume of 1600 µL, followed by the addition of 100 µL of Folin–Ciocalteu reagent (Sigma-Aldrich). After a 3 min reaction period, 300 µL of saturated sodium carbonate (Na_2_CO_3_) solution was added. The resulting mixture was incubated at 40 °C for approximately 30 min, allowing for the development of a characteristic blue coloration [49]. The optical density of the final solution was recorded at 725 nm using a spectrophotometer (HITACHI U1900, Hitachi High-Technologies Co., Tokyo, Japan). A standard calibration curve was constructed using gallic acid solutions in the concentration range of 0–2.5 mg/mL (R^2^ = 0.9994). Each kind of tannic acid formulation was tested in quadruplicate (n = 4). To maintain a constant volume of 2 mL in each well, 20 µL of fresh PBS was replenished after each sampling.

### 3.4. Cytocompatibility Evaluation

In vitro cytotoxicity tests were conducted in accordance with ISO 10993-5 standard [38] using NIH/3T3 fibroblast cells (ATCC CRL-1658) from the American Type Culture Collection (ATCC, Manassas, VA, USA) and cultured in accordance with the protocol required by the supplier. Samples were UV sterilized for 15 min on each side. In order to obtain the extract, the samples were weighed and filled with the medium in a proportion consistent with the conversion of 0.2 g/1 mL (according to the standard ISO 10993-12 [50]), taking into account that the thickness of the samples was >1.0 mm and they were made of solid material. The samples in contact with the medium were left for extraction for 24 h. After this time, the extracts were taken and added to the cell culture for the next 24 h. Fibroblast cells of the NIH/3T3 line (passage 6) were cultured in a standard culture medium (Dulbecco’s Modified Eagle’s Medium-DMEM) supplemented with 10% by volume of bovine calf serum (BCS) (ATCC, USA) and 1% by volume of antibiotics penicillin and streptomycin (P/S)) until 75% confluence was achieved, and then they were collected and seeded onto a 96-well plate in a concentration of 6 × 10^4^ cells/well. Each well was supplemented with 100 µL of culture medium, and the cells were incubated for 24 h at 37 °C and in a 5% CO_2_ atmosphere. After this time, the medium was removed, and 100 µL of extract (100%) obtained from individual samples was added to each well. The measurement was repeated 7 times for each sample (n = 7).

The cells with the extract were cultured for another 24 h, after which the MTT reagent (10 µL) was added to each well and left to incubate for 4 h at 37 °C and in a 5% CO_2_ atmosphere. After 4 h of incubation, 200 µL of DMSO was added to each well. Absorbance was measured at two wavelengths of 570 nm and 660 nm (as a reference measurement). According to the guidelines included in the standard, a positive control ((K+)—cells flooded with 100 µL of medium/well +20 µL of DMSO/well) and a negative control ((K−)—cells flooded with 100 µL of medium/well) were included. Cell viability was calculated relative to the negative control, which was 100%.

### 3.5. Antibacterial Activity

#### 3.5.1. Pre-Culture of Microorganisms and Preparation of Cell Suspensions

The studies were conducted on the reference bacteria *Escherichia coli* ATCC8739, *Staphylococcus aureus* ATCC6538P, and *Pseudomonas aeruginosa* ATCC15442. Pre-cultures of bacteria were conducted on liquid Tryptone Soy Broth medium in 100 mL Erlenmeyer flasks at 37 °C for 24 h. After this time, 2 mL was taken from the obtained cultures, and the cells were separated by a centrifugation process at a speed of 10,000 rpm. The supernatant was removed, and the cell pellet was suspended in 1 mL of sterile physiological saline. Then, the OD of the obtained suspensions was adjusted to 0.5, which corresponds to a concentration of 1.5 × 10^8^/mL. The obtained suspensions were used in the next stage of this study.

The tested discs were placed in 2 mL Eppendorf tubes. Then, the test microorganisms were added to each 1 mL of the prepared suspension of cells. The described test sets were performed in duplicate. One for testing antibacterial activity using the culture method, the other for testing using the Live/Dead staining method. The whole was left for 24 h at 22 °C.

#### 3.5.2. Antibacterial Activity Using the Culture Method

After this time, the cell suspension was collected from the Eppendorf tubes, and the procedure for determining the number of living and capable of growing cells was performed. For this purpose, 1 mL of cell suspension after contact time with the tested material was transferred to test tubes with 9 mL of sterile dilution solution (buffered peptone water with neutralizer, composition: lecithin 1 g/L). The whole was vortexed to mix. In this way, dilution 1 was prepared. From the prepared dilution 1, 1 mL was taken and transferred to another test tube with 9 mL of dilution solution, obtaining dilution 2. The procedure was repeated until dilution 4 was obtained. Then, the number of living and capable of growing cells of the tested microorganisms was determined. For this purpose, a pour Petri dish inoculation was performed on Tryptic Soy Agar medium. The inoculated plates were incubated at 30 °C for 72 h. After this time, the grown colonies were counted and their number was converted into the number of cells in the suspension after the contact time. Antibacterial activity of the tested surfaces was determined based on the determination of the degree of reduction of cells capable of growth. In accordance with the ISO 22196 standard, it was assumed that a reduction in the number of cells capable of growth by at least 2 orders of magnitude is the basis for determining the occurrence of lethality towards the tested microorganism.

#### 3.5.3. LIVE/DEAD Staining Method

After the incubation time, the cell suspension (1 mL) was transferred to a new, sterile Eppendorf tube, and 1 µL of dyes (propidium iodine and Syto 9 Green) included in the LIVE/DEATH kit (Invitrogen™ LIVE/Dead, Thermo Fisher Scientific Inc., Waltham, MA, USA) was added. After 15 min, the cell suspension was filtered through polycarbonate membrane filters with a pore diameter of 0.22 µm to retain the cells on the filter surface. Then, the filters were dried in an air stream and viewed under an epifluorescence microscope (Nikon Eclipse E200, Amstelveen, The Netherlands) connected to a DS.-Fi1 digital camera. The staining procedure causes cells with an intact cell membrane to stain green and are considered alive. On the other hand, dead cells with a damaged cell membrane stain red to orange. Microscopic images were digitally captured using the MultiScan Base v.14 image analysis program (Computer Scanning System Ltd., Warsaw, Poland).

### 3.6. Antifungal Activity

#### 3.6.1. Pre-Culture of Microorganisms and Preparation of Cell Suspensions

The study was conducted on an oral isolate of *Candida albicans*, which was obtained from the Department of Physiology and Toxicology’s own research.

The strain was identified and tested by sugar assimilation and fermentation techniques, as well as the germ tube test. The isolate was also identified using two matrix-assisted laser desorption ionization–time of flight mass spectrometry (MALDI TOF/MS). The technique is based on the analysis of protein composition (primarily ribosomal proteins) using a MALDI TOF mass spectrometer (MALDI Biotyper IVD, Bruker Daltonik GmbH, Bremen, Germany). During the analysis, the laser ionizes the proteins, and the time required for the ionized proteins to travel to the detector is determined as the mass-to-charge ratio (*m*/*z*) of the examined proteins. The signal intensity is recorded as a spectrum, which is compared with reference spectra for microorganism identification. Material from the tested colonies was analyzed in accordance with the recommendations of the MALDI TOF/MS manufacturer. The analyzed colony was placed on the analyzer plate, covered with the matrix, and read using the device’s detection. The spectra were used to identify microorganisms through MALDI Biotyper automation control and Bruker Biotyper version 4.0 software and library [51].

*Candida albicans* strain was pre-cultured on Sabouraud agar plates for 48 h at 30 °C. A culture stock solution was prepared prior to the experiment by inoculating selected colonies into sterile saline solution. The resulting suspension was adjusted to an optical density of OD = 0.3; this dilution contained 1.7 × 10^6^ cells/mL. The turbidity of the solution was measured using a DEN-1B densitometer (BioSan, Riga, Latvia). A total of 30 µL of the cell suspension was applied to Ti-TA2, Ti-TA5, and Ti-TA8 discs and incubated at 30 °C. Ti_0 discs were used as the control for this study. The described tests were performed in triplicate.

#### 3.6.2. Antifungal Activity Using the Culture Method

After 6 h and 24 h, the surfaces were carefully washed with PBS to remove unbound fungal cells and then transferred to 1 sterile saline solution. The titanium discs were washed for 5 min in an ultrasonic washer (ULTRON Electronic Equipment Works, Dywity, Poland) to detach *Candida albicans* colonies from the surface of the discs. A series of 10-fold dilutions was prepared from the resulting suspension and inoculated onto Sabouraud agar medium. The dishes were incubated for 72 h at 30 °C. After the specified incubation time, the colonies were counted and the results were expressed as colony-forming units (cfu/mL). Antifungal activity of the tested surfaces was determined based on the determination of the degree of reduction in cells capable of growth.

## 4. Conclusions

This study aimed to develop and characterize a tannic acid (TA) coating on titanium alloy and to investigate its antibacterial and antifungal efficacy. The titanium alloy surface was functionalized sequentially using low-temperature oxygen plasma treatment, followed by immersion in tannic acid solutions of 2, 5, and 8 wt%. The deposited coatings were examined in terms of morphology, chemical composition, surface wettability, and release behavior. Their cytocompatibility was also assessed, followed by antibacterial and antifungal tests.

Scanning electron microscopy (SEM) confirmed the formation of homogeneous TA coatings, while X-ray photoelectron spectroscopy (XPS) revealed dominant phenolic C–O and aromatic C–C signals, verifying the integrity of the polyphenolic layer. Water contact angle (WCA) measurements indicated a significant increase in surface hydrophilicity upon TA deposition. Compared to unmodified titanium (82.3° ± 3.6°), WCA decreased progressively from 35.2° ± 3.2° for 2% TA to 26.6° ± 2.8° for 8% TA. The Folin–Ciocalteu assay demonstrated an initial burst release of TA, followed by sustained diffusion. Coatings with 5% and 8% TA released approximately seven times more TA than the 2% coating.

Scanning electron microscopy (SEM) confirmed the formation of uniform TA coatings, while X-ray photoelectron spectroscopy (XPS) revealed dominant phenolic C–O and aromatic C–C signals, confirming the presence of a stable polyphenolic layer. Water contact angle (WCA) measurements indicated a significant increase in surface hydrophilicity upon TA deposition. Compared to unmodified titanium (82.3° ± 3.6°), WCA decreased progressively from 35.2° ± 3.2° for 2% TA to 26.6° ± 2.8° for 8% TA. The Folin–Ciocalteu assay demonstrated an initial burst release of TA, followed by sustained diffusion. Coatings with 5% and 8% TA released approximately seven times more TA than the 2% coating.

Cytocompatibility studies using NIH/3T3 fibroblast cells showed no reduction in cell viability after 24 h of exposure to 100% extracts, regardless of TA content. These results meet the requirements of the ISO 10993-5 standard [38]. Moreover, it can be seen that the coating components released into the extract (most likely including released gallic acid) enhance the proliferation of these cells.

Antibacterial activity, assessed according to ISO 22196, showed that coatings with 5% and 8% TA coatings achieved >99% reduction (≥2 log_10_) in *E. coli*, *S. aureus*, and *P. aeruginosa* population after 24 h, while the 2% coating exhibited only moderate effects. LIVE/DEAD staining corroborated bacterial membrane disruption on surfaces coated with ≥5% TA. Similarly, antifungal testing against *Candida albicans* demonstrated significant inhibition of fungal colonization at both 6 and 24 h, with ≥2 log_10_ reductions in viable counts compared to uncoated titanium.

In summary, tannic acid coatings at concentrations ≥5 wt% applied to plasma-activated titanium alloy surfaces offer a cytocompatible and effective strategy for antimicrobial functionalization of titanium implants. This approach combines potent bactericidal and fungistatic properties and holds significant promise for reducing implant-associated infections and improving long-term clinical outcomes in orthopedic and dental applications.

## Figures and Tables

**Table 1:** XPS results: surface composition of the Ti-TA2 discs, Ti-TA5 discs, and Ti-TA8 discs.

Spectrum	eV	Assignment of Peak	Ti-TA2 at. %	Total Content	Ti-TA5 at. %	Total Content	Ti-TA8 at. %	Total Content
C 1s	284.7	C–C, C–H	31.17	67.99	30.83	67.06	42.38	74.09
	286.3	C–O	29.65		28.80		24.89	
	288.7	O–C=O	7.17		7.43		6.82	
O 1s	531.8	O=C	3.30	32.01	4.10	32.94	4.69	25.91
	533.3	C–O–C, C–OH	28.71		28.84		21.22	
Total				100.00		100.00		100.00

**Table 2:** Number of microorganism cells capable of growth after the contact time.

	Sample	Ti_0	Ti-TA2	Ti-TA5	Ti-TA8
Tested Bacteria					
*E. coli*		1.7 × 10^8^	5.0 × 10^7^	9.0 × 10^5^	<1.0 × 10^4^
*S. aureus*		1.8 × 10^8^	4.0 × 10^7^	8.0 × 10^5^	1.0 × 10^4^
*P. aeruginosa*		1.9 × 10^8^	3.8 × 10^7^	4.0 × 10^5^	1.0 × 10^5^

**Table 3:** Number of *Candida albicans* cells capable of growth after the contact time.

Incubation Time	*C. albicans*	Ti_0	Ti-TA2	Ti-TA5	Ti-TA8
6 h	A	5.2 × 10^4^	5.1 × 10^4^	1.1 × 10^4^	2.3 × 10^4^
	B	6.0 × 10^4^	2.7 × 10^4^	6.5 × 10^3^	6.5 × 10^3^
	C	5.6 × 10^4^	1.4 × 10^4^	1.2 × 10^4^	1.2 × 10^4^
	Mean (A–C)	5.6 × 10^4^	3.1 × 10^4^	1.0 × 10^4^	1.3 × 10^4^
24 h	A	5.1 × 10^5^	1.4 × 10^6^	2.1 × 10^3^	6.7 × 10^3^
	B	5.5 × 10^5^	1.6 × 10^6^	3.9 × 10^3^	9.4 × 10^3^
	C	5.2 × 10^5^	2.1 × 10^6^	2.9 × 10^3^	8.8 × 10^3^
	Mean (A–C)	5.3 × 10^5^	1.7 × 10^6^	3.0 × 10^3^	8.3 × 10^3^

## Data Availability

The data presented in this study are available upon request from the corresponding author.
